# Supplementary material for: Sex-specific and pleiotropic effects underlying kidney function identified from GWAS meta-analysis
Source: Nat Commun. 2019 Apr 23;10:1847. doi: 10.1038/s41467-019-09861-z (PMC6478837; doi:10.1038/s41467-019-09861-z)
Supplement: Supplementary file 1 — Supplementary Information [file 41467_2019_9861_MOESM1_ESM.pdf]

Supplementary Information:

Graham et al. **“**Sex-specific and pleiotropic effects underlying kidney function identified from GWAS meta-analysis”

Supplementary Figure 1:

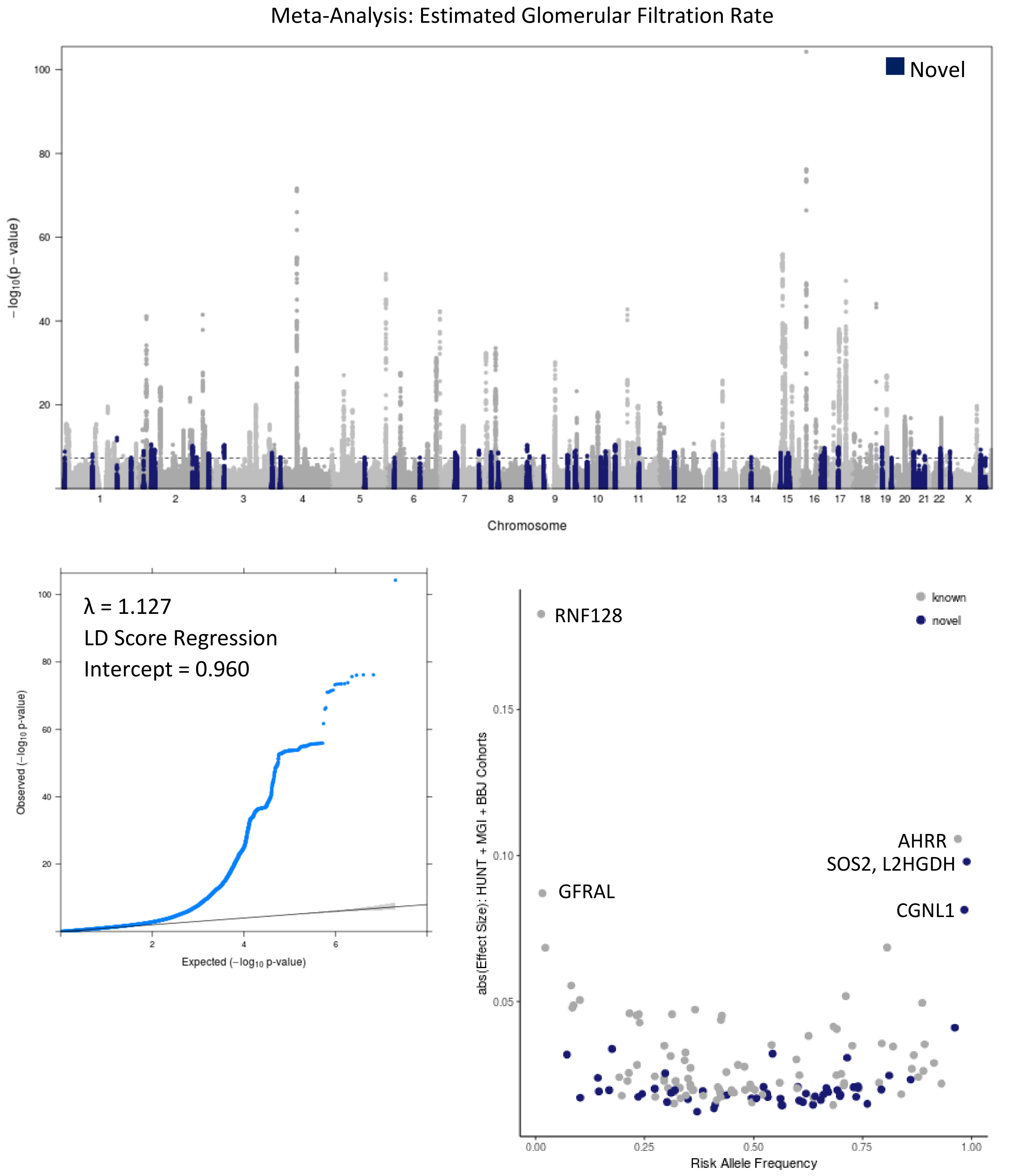

Manhattan and QQ Plot (MAF > 0.5%) from meta-analysis of eGFR. The effect sizes (from meta-analysis of HUNT, MGI, and BBJ cohorts due to differing units of CKDGen consortium) are plotted against the risk allele frequencies for the index variants from the overall meta-analysis. Prioritized genes are labeled for variants with the largest effect sizes.

Supplementary Figure 2

CKD effect size vs. eGFR effect size

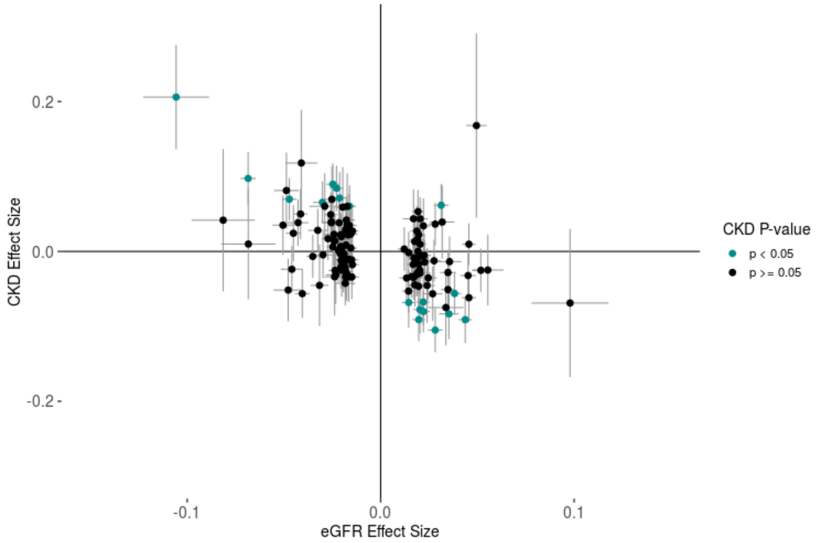

Hypertension effect size vs. eGFR effect size

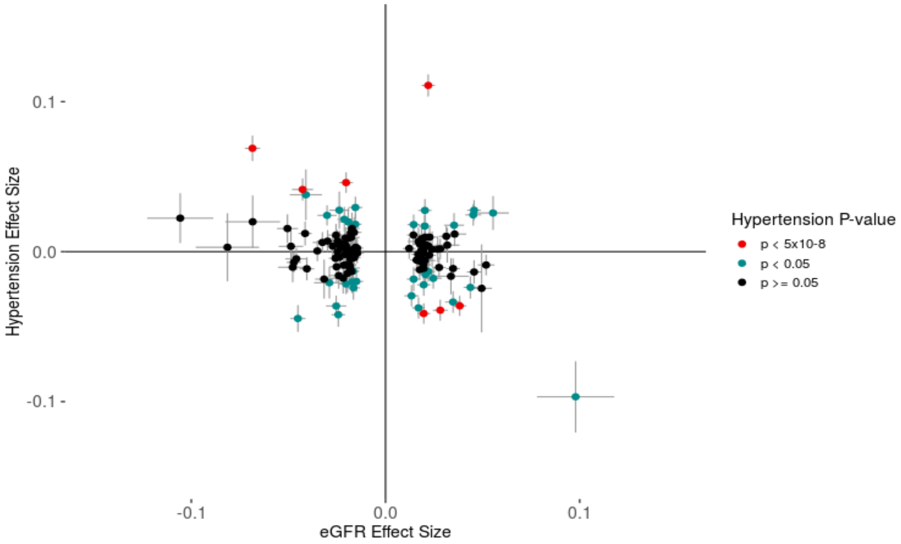

Comparison of eGFR and CKD or hypertension effect sizes for eGFR index variants. The plots are colored according to the significance of the variants for either CKD or hypertension. Error bars represent ± 1 SE.

Supplementary Figure 3

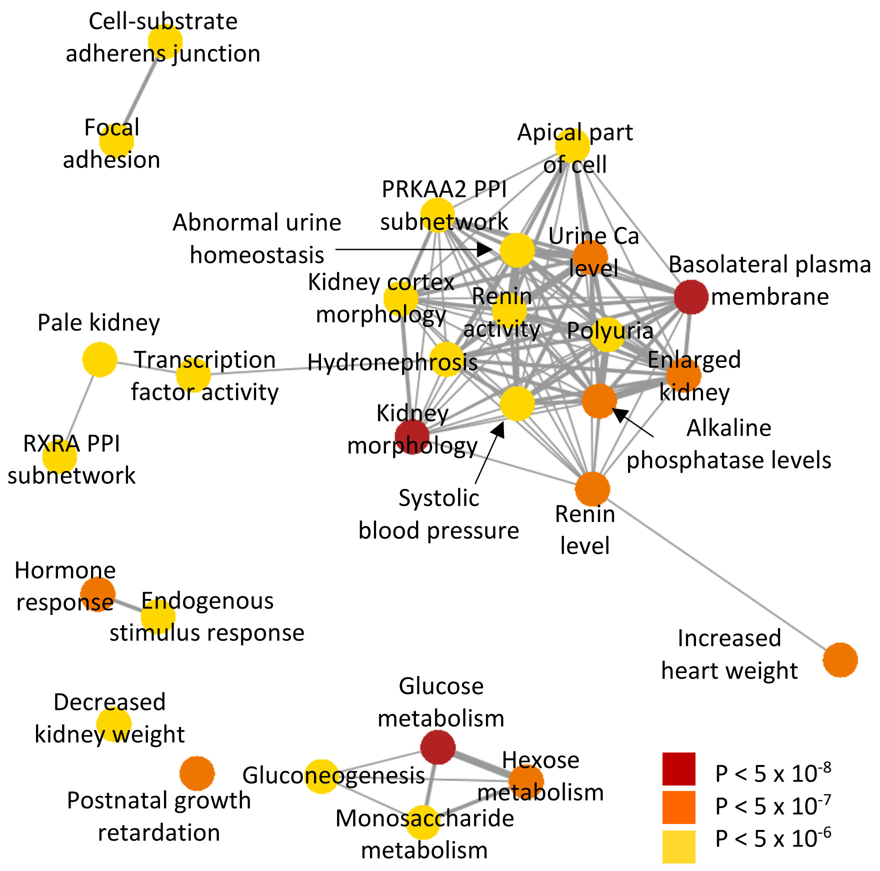

DEPICT network graph prior to collapsing overlapping gene sets. Overlap between gene sets is depicted by the width of connecting lines.

Supplementary Figure 4

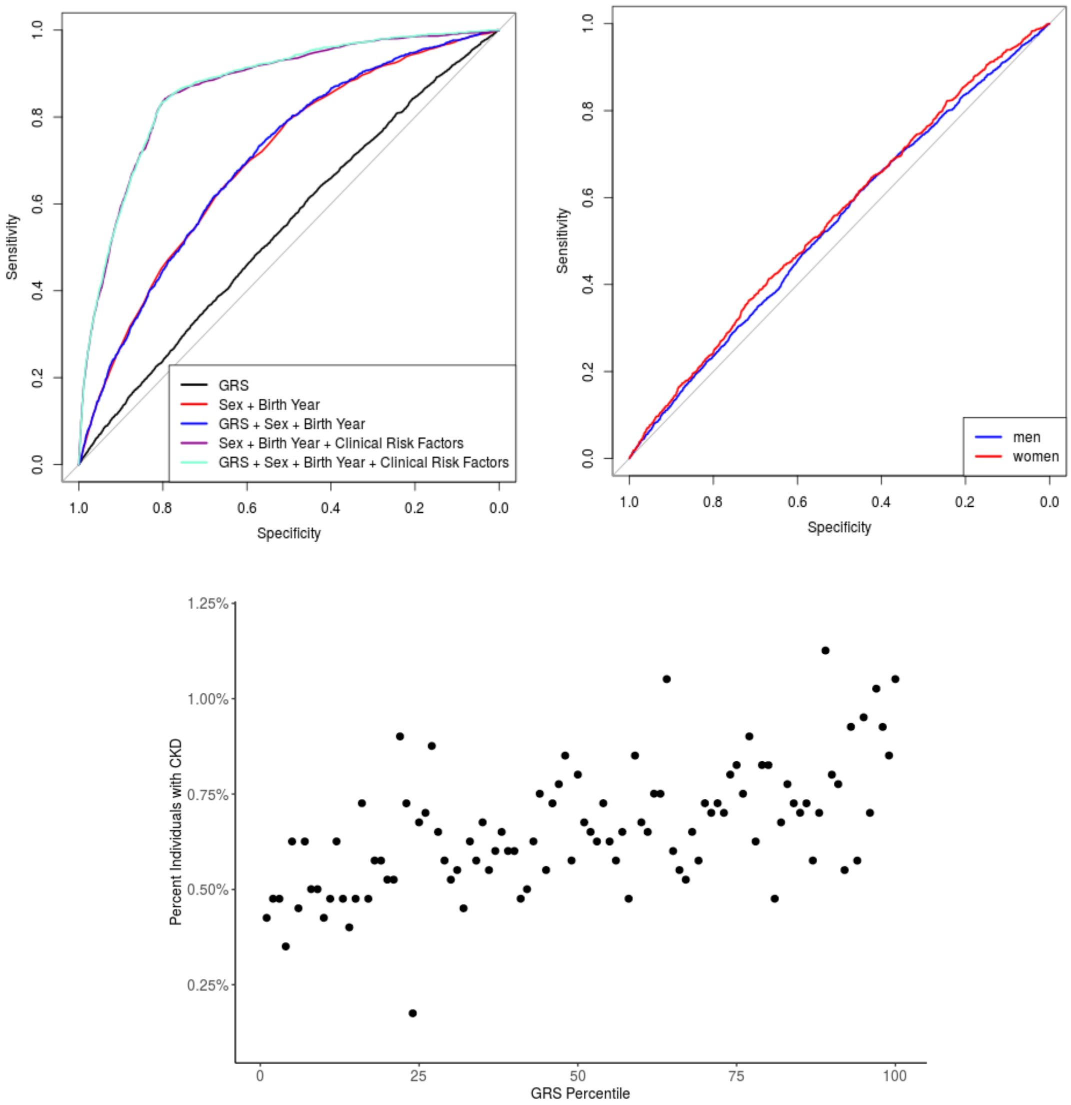

ROC plots for eGFR genetic risk scores as predictors of CKD in the white British subset of UK Biobank. Plots are shown for the best-performing genetic risk score taken from clumping of the eGFR meta-analysis results using the European subset of 1000 Genomes and all independent markers ($r^2 < 0.4$) with p-value < $5x10^{-6}$. CKD prevalence in the highest GRS percentile (1.05%) was 2.5 times higher than that of the lowest GRS percentile (0.43%) and 1.6 times higher than CKD prevalence among all other percentiles (0.65%).

Supplementary Figure 5

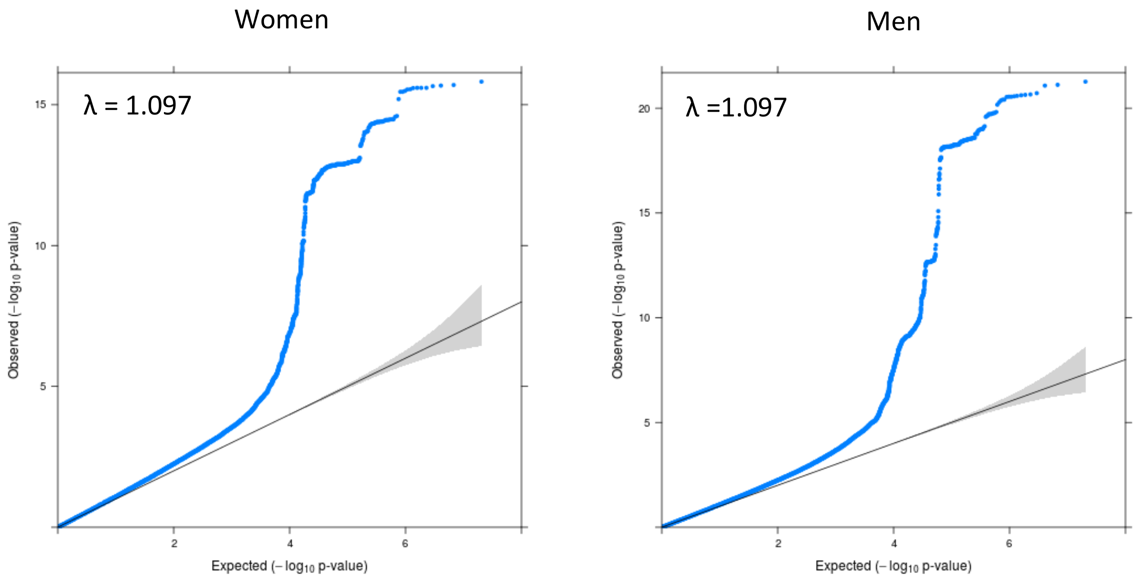

QQ Plots from sex-specific analysis for variants with MAF > 0.5%

Supplementary Figure 6

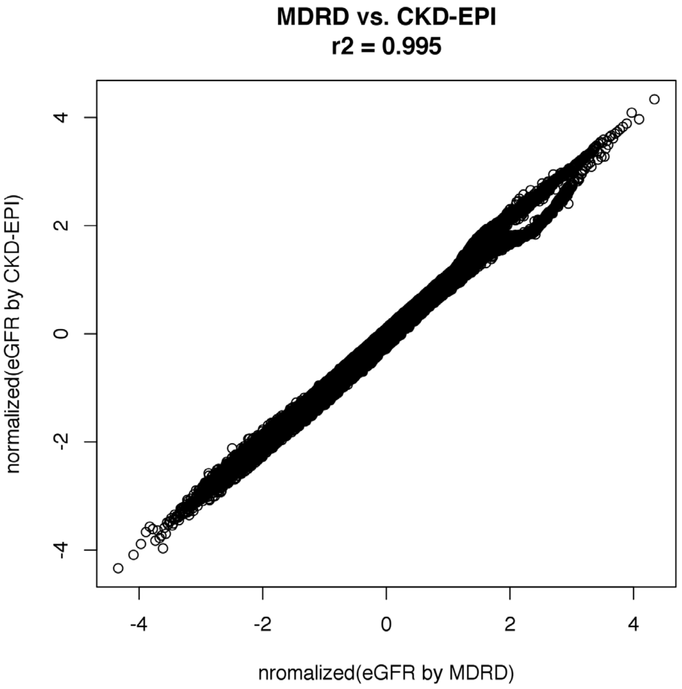

Comparison of eGFR phenotype values calculated with MDRD and CKD-EPI equations after adjustment for covariates

Supplementary Figure 7

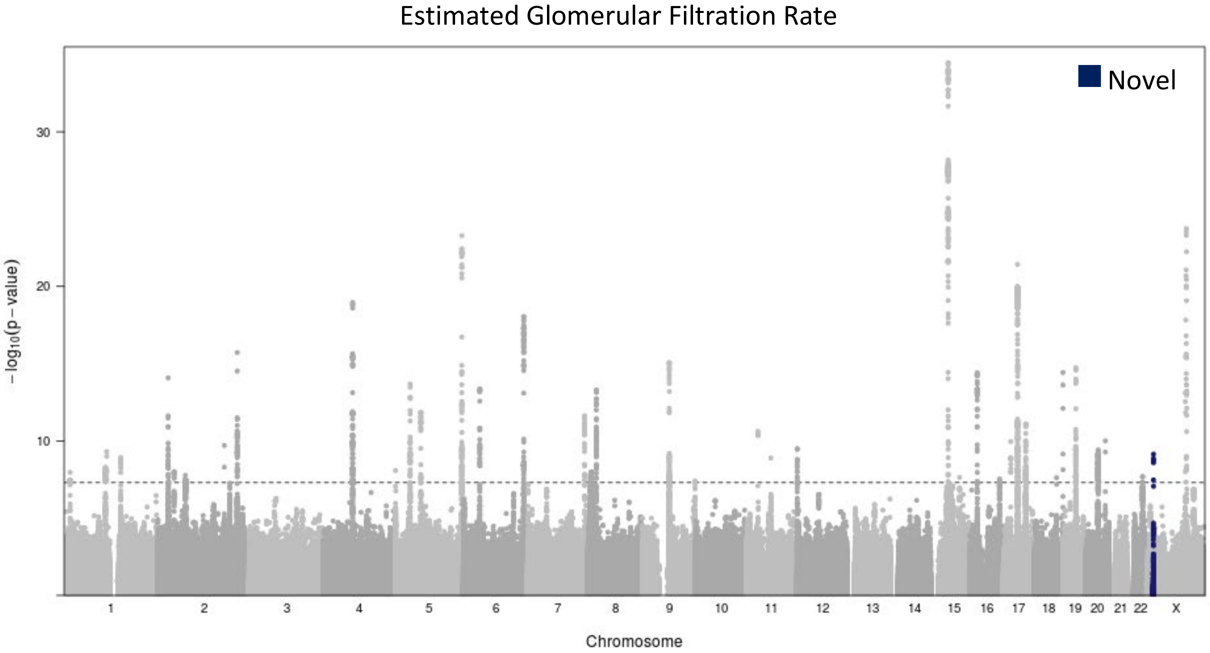

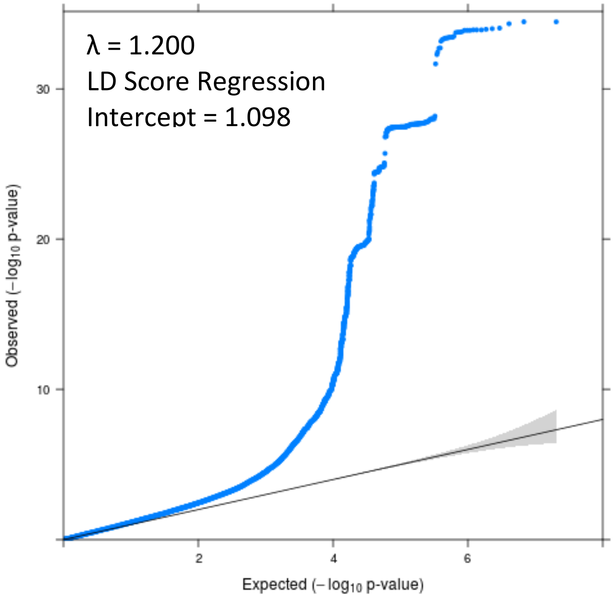

Manhattan and QQ Plot (MAF > 0.5%) from HUNT analysis of eGFR

Supplementary Figure 8

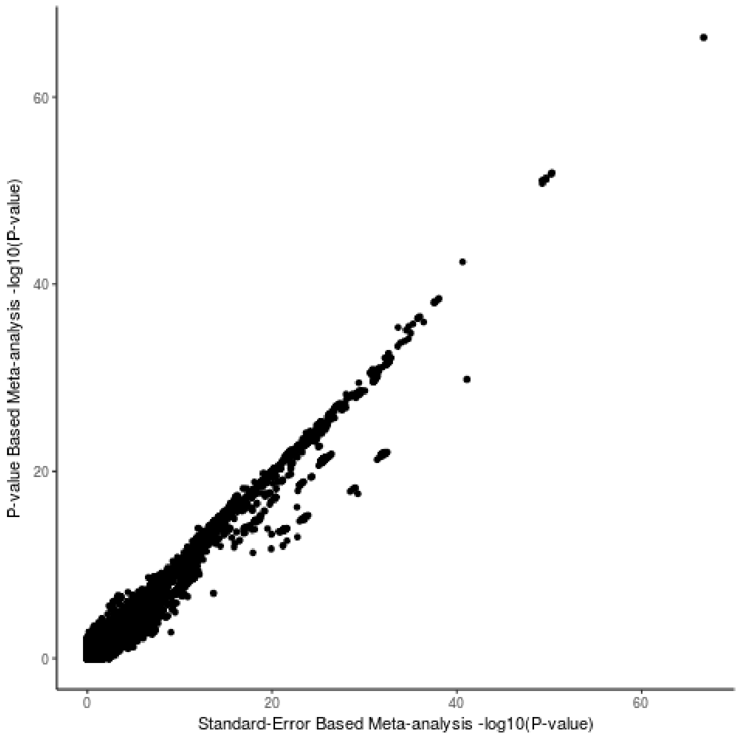

Meta-analysis of the HUNT, BBJ, and MGI cohorts was performed using both p-value and standard-error-based approaches. The overall correlation in p-values for individual variants between methods was extremely high (Pearson r = 0.966). P-values obtained using the standard-error-based approach trended towards increased significance for a subset of variants. This is likely due to higher variability in allele frequencies than average for these variants between European (HUNT and MGI) and East Asian (BBJ) ancestries. Variants reaching genome-wide significance were very similar between methods.

Supplementary Figure 9

eQTL Data Sources

GTEx v7 (48 tissue types available in public single-tissue cis-eQTL dataset, $N_{samples}$= 80-491)

Kidney Cortex (Ko et al., $N_{samples}$ = 96)

Kidney Glomerulus (NephQTL, $N_{samples}$ = 136)

Kidney Tubulointerstitium (NephQTL, $N_{samples}$ = 166)

↓ 51 tissue types

eQTL Associations

All 147 eGFR index variants were assessed for association in the eQTL datasets

Significance threshold = p-value < 6.7×10^{-6}
(Bonferroni correction for 51 tissue types and 147 index variants)

↓ eQTL associations for:
147 eGFR index variants
51 tissue types

Kidney eQTL Associations

16 genes had expression levels associated with eGFR index variants in kidney cortex, glomerulus, or tubulointerstitium

Significance threshold maintained as p-value < 6.7×10^{-6}
(Bonferroni correction for 51 tissue types and 147 index variants)

5 of these 16 genes were significant eQTLs only in kidney tissues
(p-value for gene in non-kidney tissues > 6.7×10^{-6})

eQTL datasets from GTEx V7, NephQTL, and Ko et al. were used to identify eGFR index variants which may be acting through the regulation of gene expression. The following flowchart summarizes the included samples and datasets. Complete listings of tissue types, data sources, and sample sizes are given in **Supplementary Table 2**.

Supplementary Table 1: Significant missense variants from meta-analysis of eGFR

Chr	Pos (hg19)	Ref	Alt	rsID	Freq	FreqSE	Weight	Zscore	pvalue	Direction	Gene	Protein Variants	r^2 with lead
1	15808767	A	G	rs3820071	0.6	0.1153	347323	5.995	2.03E-09	++++	CELA2B	p.Gly79Arg	0.6142
1	15808872	A	G	rs3766160	0.612	0.1111	347323	5.622	1.88E-08	++++	CELA2B	p.Asp114Asn	0.6365
1	113255456	A	C	rs34611728	0.9163	0.0324	206846	8.054	8.01E-16	++?+	PPM1J	p.Leu213Phe	0.9828
1	150737220	A	G	rs41271951	0.0586	0.0236	206846	6.662	2.71E-11	++?+	CTSS	p.Val7Ala	0.2995
1	150940625	T	G	rs267738	0.1075	0.0674	347323	9.238	2.52E-20	++++	CERS2	p.Glu115Val	lead variant
1	150958836	A	G	rs267733	0.0856	0.0448	347322	6.105	1.03E-09	++++	ANXA9	p.Asp166Gly	0.5489
1	150974971	A	T	rs41310885	0.9351	0.0046	206846	-6.365	1.96E-10	--?-	FAM63A	p.Arg41Ser	0.2561
1	151015868	A	G	rs12068365	0.9309	0.0053	203665	-5.818	5.94E-09	--?-	BNIPL	p.Ser226Asn	0.2337
1	151062957	A	G	rs11204774	0.9351	0.0068	203662	-5.922	3.18E-09	--?-	GABPB2	p.Val62Ile	0.2337
1	155178782	A	T	rs760077	0.7098	0.1262	350504	6.173	6.69E-10	++++	MTX1	p.Ser63Thr	0.9725
1	171509327	A	G	rs760644	0.1958	0.1854	350504	-6.325	2.54E-10	----	PRRC2C	p.Ala906Thr	0.7490
1	171514731	C	G	rs235468	0.8066	0.1854	350504	6.319	2.63E-10	++++	PRRC2C	p.Ser1624Cys	0.7492
1	201016296	A	G	rs3850625	0.931	0.0343	347322	-5.66	1.52E-08	----	CACNA1S	p.Arg1539Cys	lead variant
1	243493907	A	T	rs2275155	0.2286	0.0541	350504	-6.159	7.30E-10	----	SDCCAG8	p.Glu378Asp	0.5011
2	27730940	T	C	rs1260326	0.5403	0.1034	347318	-13.546	8.34E-42	----	GCKR	p.Leu446Pro	0.9097
2	27801493	T	C	rs1919127	0.4139	0.1456	347319	10.012	1.36E-23	++++	C2orf16	p.Val685Ala	0.4367
2	27801759	A	G	rs1919128	0.405	0.1492	350504	10.042	1.00E-23	++++	C2orf16	p.Ile774Val	0.4367
2	27851918	A	G	rs3749147	0.7104	0.0759	350504	-8.422	3.69E-17	----	GPN1	p.Arg12Lys	0.2890
2	54587596	T	C	rs2280718	0.2865	0.152	347320	-5.913	3.35E-09	----	C2orf73	p.Pro254Leu	0.9636
2	73651967	T	C	rs3813227	0.8279	0.1394	347322	-7.346	2.05E-13	--+-	ALMS1	p.Arg393Cys	0.8587
2	73677898	C	G	rs6546837	0.8265	0.1395	350504	-7.413	1.24E-13	--+-	ALMS1	p.Gly1416Ala	0.8587
2	73679280	A	G	rs6546838	0.1711	0.1381	347323	7.287	3.16E-13	++-+	ALMS1	p.Ile1877Val	0.8546
2	73679866	T	C	rs10496192	0.1262	0.025	206846	8.561	1.12E-17	++?+	ALMS1	p.Ile2072Thr	0.6323
2	73679990	A	T	rs6724782	0.8266	0.1394	350504	-7.383	1.54E-13	--+-	ALMS1	p.Ser2113Arg	0.8546
2	73680508	C	G	rs6546839	0.8266	0.1394	350504	-7.384	1.54E-13	--+-	ALMS1	p.Arg2286Pro	0.8546
2	73717567	T	G	rs2056486	0.8293	0.1379	347322	-7.332	2.28E-13	--+-	ALMS1	p.Arg2828Ser	0.8587
2	73717656	A	G	rs10193972	0.1709	0.1379	347731	7.336	2.20E-13	++-+	ALMS1	p.Asn2858Ser	0.8587
2	73828538	A	G	rs1052161	0.3688	0.0952	347322	8.837	9.85E-19	++++	ALMS1	p.Arg4031Lys	0.2868
2	73868328	A	G	rs13538	0.2315	0.0215	206846	10.176	2.55E-24	++?+	NAT8	p.Phe143Ser	0.9845

2	74007136	T	C	rs2272051	0.6287	0.0861	347322	-7.168	7.63E-13	----	DUSP11	p.Asp36Gly	0.1699
2	148716428	T	C	rs2307394	0.3991	0.0796	347320	-7.184	6.77E-13	----	ORC4	p.Asn78Thr	0.8890
2	211540507	A	C	rs1047891	0.7685	0.0682	350504	13.619	3.11E-42	++++	CPS1	p.Thr1412Asn	lead variant
3	121643804	T	C	rs2257212	0.4184	0.1226	347323	7.485	7.13E-14	++++	SLC15A2	p.Leu350Phe	1
3	121647286	T	C	rs1143671	0.4184	0.1226	347323	7.499	6.42E-14	++++	SLC15A2	p.Pro409Ser	1
3	121648168	A	G	rs1143672	0.419	0.1224	349675	7.349	2.00E-13	++++	SLC15A2	p.Arg509Lys	1
3	185331165	A	C	rs6762208	0.6357	0.022	350504	6.846	7.57E-12	++++	SENP2	p.Thr301Lys	0.9954
3	186445052	T	G	rs2304456	0.1202	0.0226	349263	-5.825	5.70E-09	----	KNG1	p.Ile197Met	0.2989
4	77192868	A	G	rs3733250	0.6145	0.0284	347322	8.713	2.97E-18	++++	FAM47E	p.Glu273Lys	0.1991
5	39364554	A	G	rs700233	0.7412	0.1097	347318	8.415	3.93E-17	++++	C9	p.Arg5Trp	0.3496
6	31237862	T	C	rs9264621	0.1118	0.0215	301059	5.453	4.97E-08	++++	HLA-C	p.Glu299Gly	1
6	133789728	A	G	rs9493627	0.647	0.0289	347320	-5.839	5.24E-09	----	EYA4	p.Gly277Ser	0.1963
6	160670282	A	C	rs316019	0.8244	0.2091	347322	-10.636	2.03E-26	----	SLC22A2	p.Ser270Ala	0.0153
7	77552127	A	G	rs848486	0.3438	0.1064	350504	-7.96	1.73E-15	----	PHTF2	p.Arg350His	1
9	34107505	T	C	rs11557154	0.6536	0.184	350501	-5.57	2.54E-08	----	DCAF12	p.Arg131Gln	0.9936
10	1065710	T	C	rs1044261	0.9506	0.0114	203665	7.45	9.31E-14	++?+	IDI2	p.Trp144Ter	0.2526
10	69926334	C	G	rs10823148	0.3677	0.097	350504	7.843	4.41E-15	++++	MYPN	p.Phe628Leu	0.7903
10	69933921	A	G	rs10997975	0.5956	0.0615	347323	-7.669	1.74E-14	----	MYPN	p.Ser691Asn	0.7801
10	69933969	A	G	rs7916821	0.6316	0.0853	347323	-8.657	4.83E-18	----	MYPN	p.Ser707Asn	0.8747
10	69959242	A	C	rs7079481	0.6178	0.078	347322	-8.682	3.88E-18	----	MYPN	p.Pro1135Thr	0.9518
10	104573017	T	G	rs284859	0.7854	0.0487	347322	-5.824	5.74E-09	----	WBP1L	p.Ala341Ser	0.9927
11	47701528	A	C	rs12286721	0.3586	0.0849	347318	-5.934	2.96E-09	----	AGBL2	p.Met67Ile	0.4533
11	47857253	T	C	rs3816605	0.3334	0.068	347320	-5.993	2.05E-09	----	NUP160	p.Thr351Ala	0.4572
11	65143892	A	G	rs34400381	0.974	0.0086	204493	-6.251	4.08E-10	--?-	SLC25A45	p.Arg285Cys	0.0001
11	65386206	C	G	rs1193851	0.224	0.0993	350503	5.613	1.99E-08	++++	PCNX3	p.Ser458Cys	0.0908
12	48723324	C	G	rs2732441	0.2445	0.0932	206844	-5.94	2.84E-09	--?-	H1FNT	p.Arg84Gly	0.9790
12	48736985	T	G	rs2732481	0.2444	0.0946	203655	-6.014	1.82E-09	--?-	ZNF641	p.Gln363Pro	lead variant
12	112375990	A	C	rs3752630	0.182	0.0285	347727	5.597	2.18E-08	++++	TMEM116	p.Cys114Gly	0.2929
15	45545478	T	C	rs11854484	0.7297	0.2067	350504	-12.178	4.09E-34	----	SLC28A2	p.Pro22Leu	0.7265
15	45554267	A	C	rs1060896	0.7217	0.2185	347322	-12.109	9.50E-34	----	SLC28A2	p.Ser75Arg	0.7899
15	45661678	A	T	rs1288775	0.3777	0.2338	350504	15.523	2.41E-54	++++	GATM	p.Gln110His	0.6419

15	45695382	A	G	rs7182723	0.4219	0.2475	350504	15.455	7.02E-54	++++	SPATA5L1	p.Arg252Gln	0.6205
15	53907948	A	G	rs17730281	0.6832	0.0663	350504	-13.182	1.12E-39	----	WDR72	p.Leu819Phe	lead variant
15	54003091	A	G	rs551225	0.6445	0.0729	349248	9.663	4.31E-22	++++	WDR72	p.Pro306Leu	0.2218
16	20410547	T	C	rs9926580	0.7506	0.0488	345095	-10.211	1.77E-24	----	PDILT	p.Ala26Thr	0.2304
17	19474875	T	C	rs111653425	0.9849	0.0031	96329	6.8	1.04E-11	?+?+	SLC47A1	p.Ala465Val	0.0077
17	34854280	A	G	rs2306590	0.6239	0.0551	347315	-5.78	7.45E-09	----	MYO19	pLeu663Phe	0.8846
17	37814080	A	G	rs1877031	0.4725	0.0847	347321	7.379	1.60E-13	++++	STARD3	p.Arg117Gln	0.2812
17	37879588	A	G	rs1136201	0.1768	0.0568	342042	-7.303	2.82E-13	----	ERBB2	p.Ile655Val	0.0368
17	37884037	C	G	rs1058808	0.5249	0.0913	350502	-5.927	3.09E-09	---+	ERBB2	p.Pro1170Ala	0.2158
17	37944519	C	G	rs112301322	0.0281	0.0126	205584	-5.539	3.04E-08	--?-	IKZF3	p.Gly234Ala	0.0054
17	59489893	T	C	rs9907379	0.6805	0.1406	350502	11.923	9.03E-33	++++	C17orf82	p.Leu186Pro	0.1368
19	33281924	A	G	rs11881633	0.7031	0.1596	347320	6.757	1.41E-11	++++	TDRD12	p.Lys413Glu	0.1569
19	33293607	T	C	rs11673502	0.9016	0.0328	350504	-6.859	6.95E-12	----	TDRD12	p.Thr748Met	0.1180
19	33301074	A	G	rs144485154	0.0174	0.0103	195378	5.568	2.58E-08	++?+	TDRD12	p.Thr915Ala	0.0092
19	33309030	A	T	rs12151363	0.0975	0.0335	350504	6.894	5.41E-12	++++	TDRD12	p.Gln1122Leu	0.1200
19	33414420	A	G	rs4805825	0.3782	0.1563	347323	5.892	3.81E-09	++++	CEP89	p.Val398Ala	0.4238
20	33587198	C	G	rs3746435	0.8428	0.0177	350502	5.56	2.71E-08	++++	MYH7B	p.Lys1552Asn	0.3063
20	62340115	C	G	rs1291212	0.8324	0.2939	204319	5.978	2.25E-09	++?+	ZGPAT	p.Ser61Arg	0.9772
20	62369997	T	C	rs1151625	0.9432	0.0168	201550	5.519	3.41E-08	++?+	LIME1	p.Pro211Leu	0.9659
X	106016268	A	T	rs56121637	0.0127	0.0038	96329	-9.266	1.94E-20	?-?-	RNF128	p.Asn204Tyr	lead variant
X	106221389	T	C	rs41304048	0.0268	0.0052	96329	-7.374	1.66E-13	?-?-	MORC4	p.Asn326Ser	0.0241

Supplementary Table 2: eQTL data sources with sample size

Tissue Type	Data Source	Sample Size
Adipose_Subcutaneous	GTEx v7	385
Adipose_Visceral_Omentum	GTEx v7	313
Adrenal_Gland	GTEx v7	175
Artery_Aorta	GTEx v7	267
Artery_Coronary	GTEx v7	152
Artery_Tibial	GTEx v7	388
Brain_Amygdala	GTEx v7	88
Brain_Anterior_cingulate_cortex_BA24	GTEx v7	109
Brain_Caudate_basal_ganglia	GTEx v7	144
Brain_Cerebellar_Hemisphere	GTEx v7	125
Brain_Cerebellum	GTEx v7	154
Brain_Cortex	GTEx v7	136
Brain_Frontal_Cortex_BA9	GTEx v7	118
Brain_Hippocampus	GTEx v7	111
Brain_Hypothalamus	GTEx v7	108
Brain_Nucleus_accumbens_basal_ganglia	GTEx v7	130
Brain_Putamen_basal_ganglia	GTEx v7	111
Brain_Spinal_cord_cervical_c-1	GTEx v7	83
Brain_Substantia_nigra	GTEx v7	80
Breast_Mammary_Tissue	GTEx v7	251
Cells_EBV-transformed_lymphocytes	GTEx v7	117
Cells_Transformed_fibroblasts	GTEx v7	300
Colon_Sigmoid	GTEx v7	203
Colon_Transverse	GTEx v7	246
Esophagus_Gastroesophageal_Junction	GTEx v7	213
Esophagus_Mucosa	GTEx v7	358
Esophagus_Muscularis	GTEx v7	335
Heart_Atrial_Appendage	GTEx v7	264
Heart_Left_Ventricle	GTEx v7	272
Liver	GTEx v7	153
Lung	GTEx v7	383
Minor_Salivary_Gland	GTEx v7	85
Muscle_Skeletal	GTEx v7	491
Nerve_Tibial	GTEx v7	361
Ovary	GTEx v7	122
Pancreas	GTEx v7	220
Pituitary	GTEx v7	157
Prostate	GTEx v7	132
Skin_Not_Sun_Exposed_Suprapubic	GTEx v7	335
Skin_Sun_Exposed_Lower_leg	GTEx v7	414
Small_Intestine_Terminal_Ileum	GTEx v7	122

Spleen	GTEx v7	146
Stomach	GTEx v7	237
Testis	GTEx v7	225
Thyroid	GTEx v7	399
Uterus	GTEx v7	101
Vagina	GTEx v7	106
Whole_Blood	GTEx v7	369
Kidney_Cortex	Ko et al. 2017	96
Glomerulus	NephQTL	136
Tubulointerstitium	NephQTL	166
Dataset file locations		
GTEx v7	https://gtexportal.org/home/datasets	GTEx_Analysis_v7_eQTL.tar.gz
NephQTL	https://umich.app.box.com/s/6jrvgmdh0ppwqvgriuvijiau0xcb4ohm	peerEQTL_G.2017-09-25.reformat.bgzip.cis.gz, peerEQTL_T.2017-09-25.reformat.bgzip.cis.gz
Ko et al. 2017	https://ars.els-cdn.com/content/image/1-s2.0-S000292971730191X-mmc4.xlsx	

Supplementary Table 3a: Significant missense variants from eGFR analysis in HUNT

Chr	Pos (hg19)	Ref	Alt	rsID	Freq	Beta	SE	P-value (GC)	Gene	Protein Variant	r^2 with lead
1	113255456	C	A	rs34611728	0.112406	-0.05373	0.008792	2.42E-08	PPM1J	p.Leu213Phe	0.9763
1	150940625	T	G	rs267738	0.209508	0.040973	0.00685	4.74E-08	CERS2	p.Glu115Ala	0.5462
2	27730940	T	C	rs1260326	0.676098	-0.04158	0.005934	1.59E-10	GCKR	p.Leu446Pro	0.5303
2	211540507	C	A	rs1047891	0.304447	-0.05076	0.006169	5.88E-14	CPS1	p.Thr1412Asn	lead variant
4	77192868	G	A	rs3733250	0.426614	-0.03639	0.005655	4.23E-09	FAM47E	p.Glu273Lys	0.2527
5	39364554	G	A	rs700233	0.391922	-0.03444	0.005743	4.38E-08	C9	p.Arg5Trp	0.7857
11	65143892	G	A	rs34400381	0.036074	0.090692	0.014957	3.11E-08	SLC25A45	p.Arg285Cys	lead variant
15	45545478	C	T	rs11854484	0.593482	0.057955	0.005704	1.78E-20	SLC28A2	p.Pro22Leu	0.7144
15	45554267	C	A	rs1060896	0.62986	0.06017	0.005801	2.83E-21	SLC28A2	p.Ser75Arg	0.8120
15	45661678	T	A	rs1288775	0.299525	-0.06749	0.006113	6.85E-24	GATM	p.Gln110His	0.6286
15	45695382	G	A	rs7182723	0.298607	-0.06735	0.006131	1.14E-23	SPATA5L1	p.Arg252Gln	0.6257
17	19474875	C	T	rs111653425	0.017013	-0.13982	0.021654	3.76E-09	SLC47A1	p.Ala465Val	lead variant
17	37814080	G	A	rs1877031	0.670095	-0.04025	0.005953	6.76E-10	STARD3	p.Arg117Gln	0.4534
17	37879588	A	G	rs1136201	0.276071	-0.04082	0.006248	2.47E-09	ERBB2	p.Ile655Val	0.0926
X	106016268	A	T	rs56121637	0.015023	-0.19326	0.018933	1.18E-20	RNF128	p.Asn204Tyr	lead variant
X	106221389	T	C	rs41304048	0.030001	-0.10082	0.013522	1.00E-11	MORC4	p.Asn326Ser	0.4901

Supplementary Table 3b: Stop-gain variants identified from creatinine analysis in HUNT

Chr	Pos (hg19)	Ref	Alt	rsID	Freq	Beta	SE	P-value	Gene	Protein Variant	r^2 with lead
4	88996055	C	T	rs755226061	0.000267	1.045884	0.178346	4.51E-09	PKD2	p.Arg872Ter	0

Supplementary Table 4: Significant variants from conditional analysis for eGFR in HUNT

Chr	Pos (hg19)	Ref	Alt	rsID	Freq	Beta	SE	Conditional P-value	Variants Conditioned on	Original P-value	eQTL	Nearest Gene
6	160575985	C	T	rs2297374	0.349894	-0.03521	0.005818	1.40E-09	6:160668186_CCT/C	1.10E-09	SLC22A1	SLC22A1
11	30754837	G	A	rs55733296	0.040983	-0.10866	0.01512	6.60E-13	11:30760335_T/C	8.20E-08	NA	LOC101928316, LOC101928338
17	59043487	T	G	rs72843596	0.153791	0.047923	0.007913	1.40E-09	17:59476066_A/C	1.10E-10	NA	BCAS3
