# Supplementary material for: Sex-specific and pleiotropic effects underlying kidney function identified from GWAS meta-analysis
Source: Nat Commun. 2019 Apr 23;10:1847. doi: 10.1038/s41467-019-09861-z (PMC6478837; doi:10.1038/s41467-019-09861-z)
Supplement: Supplementary file 3 — Description of Additional Supplementary Files [file 41467_2019_9861_MOESM3_ESM.pdf]

Description of Additional Supplementary Files

Supplementary data 1: Meta-analysis results for eGFR
Supplementary data 2: Gene prioritization strategy at genome-wide significant loci for eGFR
Supplementary data 3: Colocalization results of eQTL and eGFR
Supplementary data 4: DEPICT gene prioritization results
Supplementary data 5: eGFR meta-analysis results with lookup of association results for related kidney, diabetic, and cardiovascular traits in UK Biobank
Supplementary data 6: Previously reported index variants with p-value from meta-analysis with previously published datasets excluded (HUNT + MGI)
Supplementary data 7: Significant eQTL associations for index variants
Supplementary data 8: DEPICT tissue enrichment
Supplementary data 9: DEPICT gene set enrichment
Supplementary data 10: Details of kidney, cardiovascular, and other traits within UK Biobank assessed for overlap with eGFR loci
Supplementary data 11: eGFR index variants from overall meta-analysis with association results stratified by sex within HUNT
Supplementary data 12: Significant loci from HUNT association results
